# Effects of host state and body condition on parasite infestation of bent-wing bats

**DOI:** 10.1186/s12983-022-00457-w

**Published:** 2022-03-05

**Authors:** Yik Ling Tai, Ya-Fu Lee, Yen-Min Kuo, Yu-Jen Kuo

**Affiliations:** Department of Life Sciences, National Cheng Kung University, 1 University Road, Tainan, 701 Taiwan

**Keywords:** Bats, Bat flies, Body condition, *Miniopterus fuliginosus*, Parasites, Wing mites

## Abstract

**Background:**

Ectoparasites inhabit the body surface or outgrowths of hosts and are usually detrimental to host health and wellbeing. Hosts, however, vary in quality and may lead ectoparasites to aggregate on preferred hosts, resulting in a heterogeneous distribution of parasite load among hosts.

**Results:**

We set out to examine the effects of host individual state and body condition on the parasite load of multiple nycteribiid and streblid bat flies and *Spinturnix* wing mites on eastern bent-wing bats *Miniopterus fuliginosus* in a tropical forest in southern Taiwan. We detected a high parasite prevalence of 98.9% among the sampled bats, with nearly 75% of the bats harboring three or more species of parasites. The parasite abundance was higher in the wet season from mid spring to early fall, coinciding with the breeding period of female bats, than in the dry winter season. In both seasonal periods, the overall parasite abundance of adult females was higher than that of adult males. Among the bats, reproductive females, particularly lactating females, exhibited a higher body condition and were generally most infested. The *Penicillidia jenynsii* and *Nycteribia parvula* bat flies showed a consistent female-biased infection pattern. The *N. allotopa* and *Ascodipteron speiserianum* flies, however, showed a tendency towards bats of a moderate to higher body condition, particularly reproductive females and adult males.

**Conclusions:**

We found an overall positive correlation between parasite abundance and reproductive state and body condition of the host and female-biased parasitism for *M. fuliginosus* bats. However, the effects of body condition and female-biased infestation appear to be parasite species specific, and suggest that the mobility, life history, and potential inter-species interactions of the parasites may all play important roles.

## Background

Ectoparasites live on or in the skin surface of host organisms and can harm hosts by feeding on their tissues and causing irritations, or by vectoring pathogens [1]. From the perspective of parasites, however, hosts function as “habitats” providing needed space and food resources, and therefore are like habitat islands of varying individual quality [2, 3]. It seems reasonable to expect that ectoparasites would aggregate preferentially on high-quality hosts, thereby enhancing parasite success and resulting in a heterogeneous distribution of the parasite load among hosts [4, 5].

Host susceptibility to parasite infestation is determined mainly by host defense, either behaviorally (e.g., [6]) or through their immune response [7], and both may be influenced by their body condition [8, 9]. As a result, ectoparasites face a trade-off between the quality of the host as an available resource and the strength of host defense in terms of the ease of infestation [10, 11]. In particular, hosts with good body condition may offer superior resources for parasite exploitation [12], but may also be better at defending against infestation [13]. By contrast, poor-condition hosts may be more vulnerable to parasite infestation (i.e., the tasty chick hypothesis, [14]), but offer inferior or less of a favorable resource for exploitation and thus may be less attractive for parasitism [8, 15].

Bats display intricate physiological, morphological, behavioral, and ecological adaptations, and harbor diverse specialized parasites [16, 17]. Being the most gregarious of mammals with diverse social systems, many species of bats roost in colonies, where their sociality and large group size may facilitate transmission of parasites and certain zoonosses [18–20]. Furthermore, bat colonies also serve a unique role in the distribution and life history of bat ectoparasites since some arthropod parasites reproduce off-host on the roost substrates [21, 22].

While male-biased helminth infections are commonly reported in certain mammals and some other vertebrates, such as fish and birds [23, but see 24], ectoparasitic arthropod parasites appear less consistent in their selection of host sex [25]. Nonetheless, female-biased arthropod parasitism in bats is commonly observed in both temperate (e.g., [26–28]) and tropical zones (e.g., [29–31]). This bias may be attributed to the sexually different and season-specific reproductive behaviors of bats (e.g., sex-based roost segregation and parental care; [32]), or changes in the female immunocompetence and body condition during reproduction [33, 34]. This in turn may lead to seasonal variations in the infestation patterns of parasites [27, 29].

However, previous findings regarding the relationship between the body condition and parasite load of bat hosts are inconsistent. For instance, some studies reported that the ectoparasites of some European *Myotis* bats prefer hosts of higher body condition (e.g. *M. myotis* and *M. blythii*, [10]; *M. bechsteinii*, [35]), other studies found no strong correlations between the host body condition and the ectoparasite load in *M. myotis* and four other *Myotis* bats [36, 37]. One study found a negative correlation between host body condition and infestation in European common bent-wing bats *Miniopterus schreibersii* [38], while no correlation was found between ectoparasite presence and body weight across several colonies of Australasian bent-wing bats *M. orianae*. [39]. Moreover, even the ectoparasites of bats have received increasing attention, partly because of the concerns for pathogen vectors particularly in temperate areas [18, 40], few studies have investigated the parasite infestation of bats in the species-rich Indomalaya and tropical eastern Asian areas (e.g., [31]).

Accordingly, the present study set out to explore the relationships between eastern bent-wing bats *M. fuliginosus* and their ectoparasites in a tropical forest in southern Taiwan. In particular, we examined the effects of body condition and host state (e.g., age, sex, and reproductive status) on parasite load of bat flies and wing mites. Body condition indicates both the resource quality of the host and the strength of its defense against parasites [41]. We tested the hypothesis that ectoparasite infestation is influenced by body condition of the host, and predicted that bats with better body condition would exhibit a higher overall parasite load than those in a poorer condition. We further tested whether parasite infestation was associated with sex and reproductive status of the hosts. In particular, we predicted that reproductive females would be more heavily infested than bats of other states due to immune system suppression during pregnancy [33] and the enormous energetic costs of parental care [34].

## Methods

### Study area and animals

Field work was conducted in Guijijaou Experimental Forest (hereafter as the GEF forest; 20°58′ N, 120°48′ E; 200–300 m in elevation, ca. 450 ha in total area), Kenting. This area is the least-disturbed and largest reef-karst monsoon forest in Taiwan with a mean monthly temperature of just over 20 °C in the coldest months of the year and around 28 °C in the peak summer months. The forest receives 2300–2500 mm annual precipitation, the majority falling between mid-April and October. The rainfall is particularly heavy during the East Asian plum rain and typhoon seasons extending from May to September (Guijijaou Weather Station data, Taiwan Forestry Research Institute).

The eastern bent-wing bat *M. fuliginosus* (Miniopteridae) used to be considered as a subspecies of the broadly distributed common bent-wing bat *M. schreibersii* [42], and is one of the most abundant resident bats in Kenting [43]. The reported ectoparasites of *M. fuliginosus* in Taiwan include bat flies of the Nycteribiidae (*Nycteribia allotopa, N. parvula, N. formosana*, *Penicillidia jenynsii*) and Streblidae (*Ascodipteron speiserianum*) families, and wing mites (*Spinturnix psi* and *S. verutus*, Spinturnicidae; [44, 45]).

### Bat sampling and morphometric measurements

Bats were sampled over a two-year period from 2016 to 2018. We deliberately separated the wet season of April to October (corresponding to the reproductive period of the female bats) from the dry winter period of November to February. Sampling was conducted biweekly in the wet season and at least monthly in the dry season. We performed sampling at the entrance of an underground cave containing a year-round resident colony of male and female bats. The bats were sampled at dusk using a custom-made soft fine-mesh net set across a rock crevice and were collected at a rate of roughly one or two bats every five min until 25–30 bats were caught. Each bat was kept individually in a clean cotton cloth bag for later process. We distinguished adults from first-year juveniles, and determined the sex and reproductive status of the adults by their primary sexual characteristics (the penis, nipples, and mammary glands) and followed by palpation [43]. For each sampled bat, we measured the forearm length to the nearest 0.01 mm using an electronic Vernier caliper (SV-03 150, E-Base, Taiwan), and the body mass to the nearest 0.1 g using an electronic scale (JYB-500, Jin Yuan, Taiwan). All the bats were offered water and mealworms (*Tenebrio molitor*) after measuring and parasite sampling and then released on site, typically within three hours after the capture. We followed [46] for handling and care of the bats throughout the study.

### Ectoparasite sampling

We searched through the fur over the entire bat body and the head, including the wing and tail membranes. Any bat flies found were collected using forceps, individually counted, and were preserved in 75% ethanol for later identification. The species and sex of the bat flies were determined under a dissection microscope (Carl Zeiss Stemi DV4, 5–50×) using the keys provided by [44]. Wing mites were often too numerous and tiny to collect, and therefore were counted in situ without removal, but subsamples of wing mites were collected for identification following [47]. The small sizes of the flies prevented measuring their individual body mass. Consequently, we randomly selected 30 flies of each identified species from bat samples collected in the prime summer months, and oven-dried them at 50 °C for five hours. We measured the dry mass to the nearest 0.1 mg using an electronic balance (Sartorius TE214S) and then computed the mean dry mass of each bat fly species in order to estimate the total biomass of each bat fly sample. We then used the data of parasite presence and abundance to estimate prevalence (number of infested bats/total number of bats examined), and mean abundance (number of parasites/number of bats examined) and mean intensity (number of parasites/number of bats infested) [17].

### Data analysis

We obtained the body condition index (BCI) of the bats using the residuals of the body mass regression on forearm length. This index is a widely adopted measure of size-corrected mass and has several positive attributes. It focuses on variation rather than mean, it is easy to interpret, and is statistically rigorous [48, 49]. We made no attempt to infer a correlation between the computed BCI values and fat stores or any specific body composition of the animals, which may not always be well correlated subject to individual variation in response to environmental conditions [50, 51]. Pregnant females were excluded from the BCI measure due to the unpredictable effect of the developing fetus on total mass. We analyzed prevalence, mean abundance, and mean intensity of infestation for each parasite species. We found a very high parasite prevalence in our bat samples, with parasite abundances and intensities very close and the differences were nearly consistent across parasite species, so parasite abundance was solely used for later further analyses.

Unless otherwise noted, all of the data reported in this study are presented as either mean ± standard error (*SE*) or relative proportion (%) values. We conducted all of the statistical tests using STATISTICA 12 (StatSoft, Tulsa, Oklahoma) for Windows 10 with an alpha value of 0.05. We performed multivariate analysis of variance (MANOVA) tests with Pillai’s *trace* values (*V*) to examine the relationships between the bat groups of different age (adult versus juvenile), sex, and reproductive status and their body mass and forearm length. When factor effects were detected, we used Tukey’s honestly significant difference (HSD) tests to locate which particular means were significantly different [52]. The relationship of body mass and BCI values was examined by general linear regression. A chi-square test was used to examine whether the proportions of bats harboring different number of parasite species deviated from the randomness. In the wet season, pregnant, lactating, and post-lactating females were grouped as reproductive females. We found no significant differences in the BCIs of the males with swollen testes (indicating spermatogenesis) and those without reproductive status. We thus grouped the males into a single group in both seasons for the analyses. For each seasonal period, we examined the effects of host state (i.e., age, sex, reproductive status) and body condition on parasite abundance using a generalized linear model (GLM) via a link function to account for the typically non-normal distribution of the parasite data [53]. The data were examined via a likelihood-ratio statistic [54], asymptotically approximated by a chi-squared distribution [52, 54], to assess the goodness of fit for the model.

## Results

### Body mass and body condition of bats

We sampled a total of 998 bats from different age, sex, and reproductive states. Bat groups of different states differed in body mass (Pillai’s *trace* *V* = 0.624, *F*_12, 1980_ = 44.0, *p* < 0.001). Overall, pregnant females were the heaviest, followed by lactating females, post-lactating females, males, non-reproductive females, and juveniles (Table 1). Little difference was observed in the forearm lengths of the bats with different states; however, juveniles and post-lactating females showed a slightly shorter length (Table 1). The BCI values of the bats were strongly correlated to the body mass (*r* = 0.97, *p* < 0.001), where lactating females showed the highest BCI, followed by post-lactating females and males, then non-reproductive females but with a broad range of variation, and juveniles (Table 1).

**Table 1:** Mea (± SE) body mass (g), forearm length (mm), BCI values (95% CI), and mean (± SE) ectoparasite abundance (number of parasites/number of bats examined) in *Miniopterus fuliginosus* in Guijijaou Experimental Forest, Kenting, Taiwan

Bat group^*^	Body mass	Forearm length	BCI	Parasite abu
J (*n* = 118)	9.8 ± 0.07^d^	45.99 ± 0.08^b^	− 0.35 (− 0.52, − 0.18)^d^	5.3 ± 0.35
AMNR (*n* = 249)	10.4 ± 0.05^c^	46.38 ± 0.06^a^	0.32 (0.19, 0.45)^bc^	6.9 ± 0.32
AMT (*n* = 100)	10.3 ± 0.06^c^	46.46 ± 0.09^a^	0.17 (0.01, 0.33)^c^	5.0 ± 0.39
AFNR (*n* = 341)	9.8 ± 0.04^d^	46.29 ± 0.05^a^	− 0.14 (− 0.73, 0.46)^d^	6.5 ± 0.29
AFP (*n* = 58)	12.1 ± 0.14^a^	46.12 ± 0.12^b^	–	11.3 ± 0.90
AFL (*n* = 56)	10.9 ± 0.08^b^	46.29 ± 0.12^a^	0.99 (0.81, 1.16)^a^	16.2 ± 0.99
AFPL (*n* = 76)	10.4 ± 0.06^c^	45.94 ± 0.10^b^	0.49 (0.35, 0.64)^b^	12.9 ± 0.75

^*^ J: juvenile, A: adult, F: female, M: male, P: pregnant, L: lactating, PL: post lactating, NR: non reproductive, T: testis swollen; –- Excluded from the analysis

Values with the same superscript letter and within the same category are not significantly different

### Parasite loads and variation

We identified five ectoparasites on the sampled bats, including three species of nycteribiid bat flies (*P. jenynsii*, *N. allotopa*, and *N. parvula*), one species of streblid fly (*A. speiserianum*) whose females embedded as cysts under the skin surface at the back of the host’s ear shell, and wing mites (*Spinturnix* spp.). Parasites were found on 987 of the sampled bats, giving an overall prevalence of 98.9%. Only 46 bats (4.6%) were infested by single parasite species, whereas nearly 75% of the bats harbored three or more types of parasites (*χ*^2^ = 466.57, *d.f*. = 5, *p* < 0.001). Among the identified parasites, the *Spinturnix* wing mites showed the highest prevalence, mean abundance and intensity, followed by the *N*. *parvula* bat flies (mean mass = 0.1 mg). The remaining bat flies (*P. jenynsii*, 0.7 mg; *N. allotopa*, 0.1 mg; *A. speiserianum,* 1.1 mg) had lower prevalence (near or less than 50%) and mean abundances (0.7–1.6), and showed mean intensities in the range of 1.7 ~ 2.6 (Table 2).

**Table 2:** Prevalence (%), mean (± SE) abundance and mean (± SE) intensity of ectoparasites in *Miniopterus fuliginosus* (*n* = 998) in Guijijaou Experimental Forest, Kenting, Taiwan

	Prevalence	Abundance	Intensity
*P. jenynsii*	59.5	1.6 ± 0.08	2.6 ± 0.11
*N. allotopa*	52.2	1.1 ± 0.05	2.0 ± 0.07
*N. parvula*	88.3	4.3 ± 0.12	4.8 ± 0.13
*A. speiserianum*	42.3	0.7 ± 0.03	1.7 ± 0.04
*Spinturnix* spp	90.7	11.2 ± 0.64	12.2 ± 0.69

### Effects of host state and body condition on parasite abundance

Lactating females harbored the highest mean parasite abundance, followed by pregnant and post-lactating females, then non-reproductive adults and juveniles (Table 1). Overall, we found that the bats with higher BCI contained higher abundance of all the parasites (GLM, *A*. *speiserianum*: χ^2^ = 18.4; *N. allotopa*: χ^2^ = 9.41; *N. parvula*: χ^2^ = 9.23; *Spinturnix* mites: χ^2^ = 15.2, all *p* values < 0.01) except the *P. jenynsii* bat flies (χ^2^ = 0.09, *p* > 0.9). We similarly detected that the female bats harbored higher abundance of all the parasites (*N. allotopa*: χ^2^ = 9.41; *N. parvula*: χ^2^ = 9.23; *P. jenynsii*: χ^2^ = 9.23; *Spinturnix* mites: χ^2^ = 15.2, all *p* values < 0.05) with the exception of the *A. speiserianum* bat flies (χ^2^ = 0.86, *p* > 0.3).

**Fig. 1:**
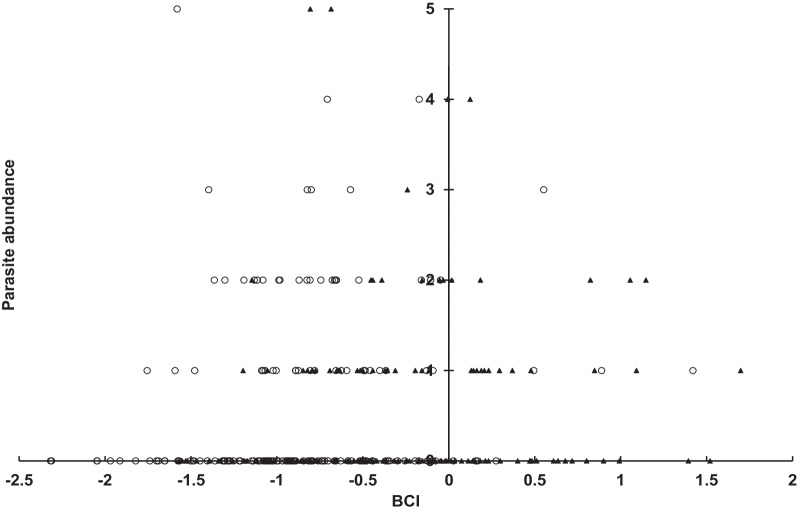
Abundance distribution of *Ascodipteron speiserianum* bat fly in relation to BCI values of female (open circle) and male (filled triangle) bent-winged bats (χ^2^ = 13.73, *p* < 0.001) in the dry season in Guijijaou Experimental Forest (hereafter as the GEF forest), Kenting, Taiwan

**Fig. 2:**
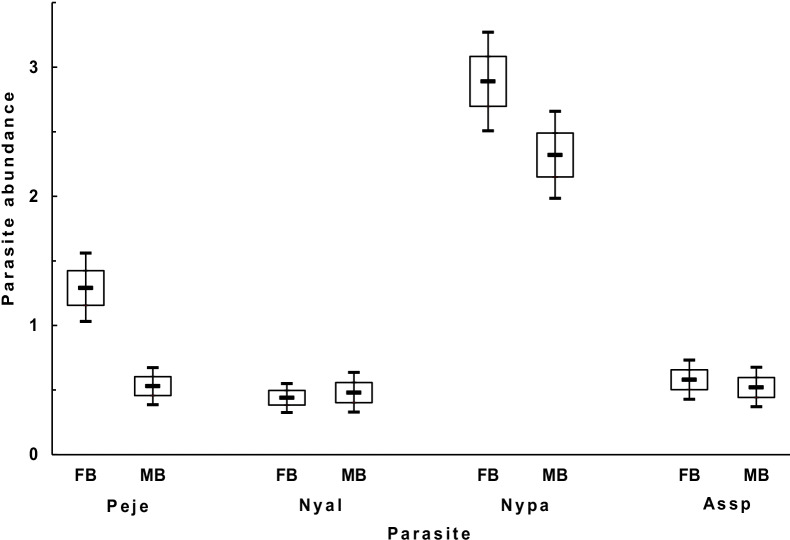
Mean (± SE; the top and bottom of the empty bar) abundance and the 95% confidence intervals (the top and bottom endpoints of the vertical line) of four species of bat flies on female (FB) and male (MB) bent-winged bats in the dry season in the GEF forest, Kenting, Taiwan. Peje: *Penicillidia jenynsii*, Nyal: *Nycteribia allotopa*, Nypa: *Nycteribia parvula*, Assp: *Ascodipteron speiserianum*

In the dry winter season from November to February, the parasite abundance of *A*. *speiserianum* was distributed convexly with the BCI for both female and male bats, and peaked around the medium BCI in both cases (Fig. 1). We also found a significant effect of the bat sex on the infestation of *P. jenynsii* (χ^2^ = 33.77, *p* < 0.001) and *N. parvula* (χ^2^ = 8.06, *p* < 0.005) flies. The female bats showed a greater mean abundance of *P. jenynsii* and *N. parvula* than the males (Fig. 2). This effect, however, was not observed for *N. allotopa* (χ^2^ = 1.18, *p* = 0.28) nor *A*. *speiserianum* (χ^2^ = 3.74, *p* = 0.053) flies. In addition, both the BCI (χ^2^ = 0.01, *p* = 0.94) and bat sex (female: 2.93 ± 0.26, CI [2.42, 3.43]; male: 2.58 ± 0.24, CI [2.12, 3.04]; χ^2^ = 2.47, *p* = 0.12) showed no effect on the mite abundance.

**Fig. 3:**
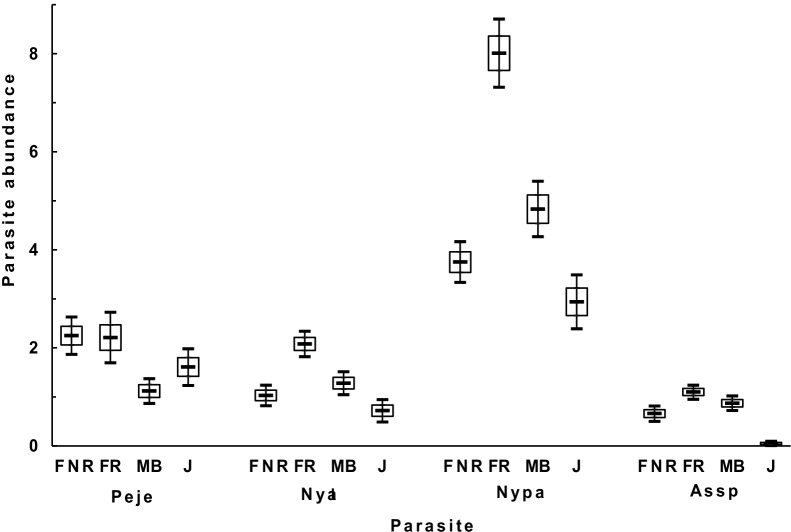
Mean (± SE; the top and bottom of the empty bar) abundance and the 95% confidence intervals (the top and bottom endpoints of the vertical line) of four species of bat flies on non-reproductive female (FNR), reproductive female (FR), male (MB), and juvenile (J) bent-winged bats in the wet season in the GEF forest, Kenting, Taiwan. Peje: *Penicillidia jenynsii*, Nyal: *Nycteribia allotopa*, Nypa: *Nycteribia parvula*, Assp: *Ascodipteron speiserianum*

In the wet season, corresponding to the breeding period of female bats and the emergence of flying juveniles in late summer, reproductive females showed the highest mean abundance of *P. jenynsii* (χ^2^ = 32.88, *p* < 0.001), *N. allotopa* (χ^2^ = 41.27, *p* < 0.001), *N. parvula* (χ^2^ = 99.03, *p* < 0.001), and *A*. *speiserianum* bat flies (χ^2^ = 104.83, *p* < 0.001; Fig. 3) and mites (23.63 ± 2.28, CI [19.13, 28.73]; χ^2^ = 174.78, *p* < 0.001). Non-reproductive females showed a similar mean abundance of *P. jenynsii* as did reproductive females, but lower abundance of *N. parvula* than did males (Fig. 3). The juveniles typically harbored the lowest abundance of bat flies, except that with greater mean abundance of *P. jenynsii* than that of the males (Fig. 3). In addition, the mite abundance on juveniles (19.46 ± 1.59, CI [16.30, 22.62]) was higher than that on males (7.21 ± 0.76, CI [5.71, 8.72]) and non-reproductive females (8.99 ± 1.17, CI: [6.69, 11.29]).

**Fig. 4:**
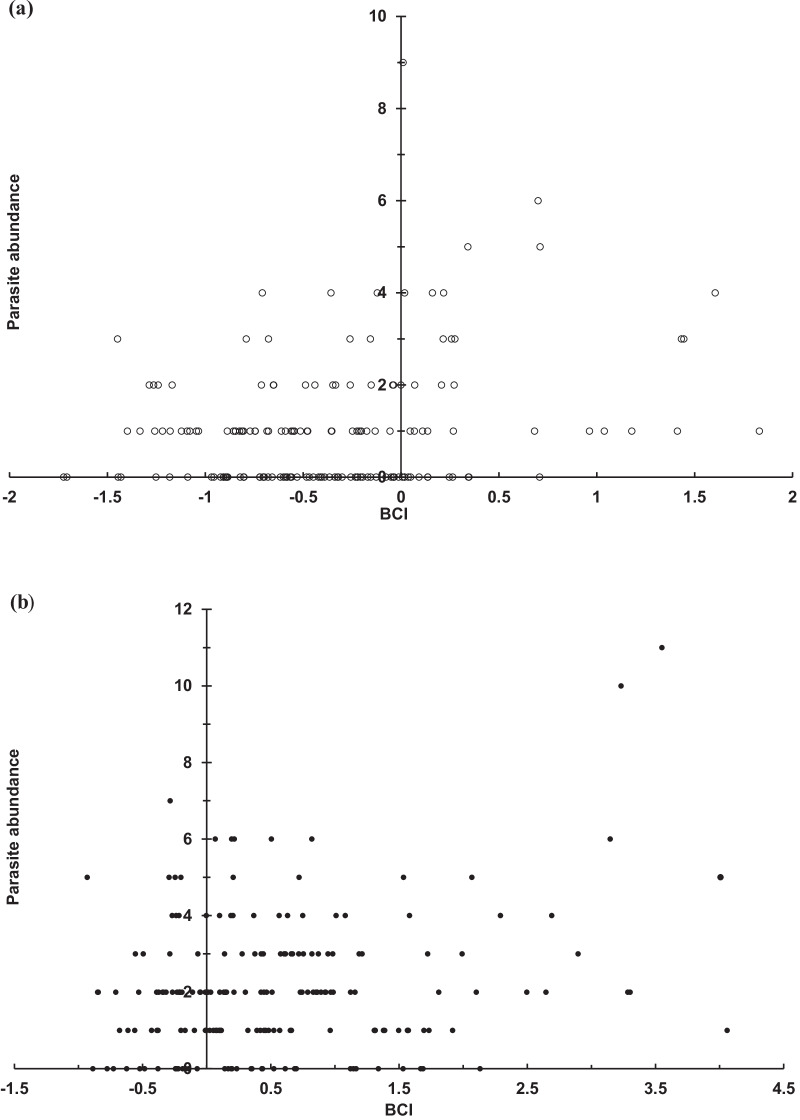

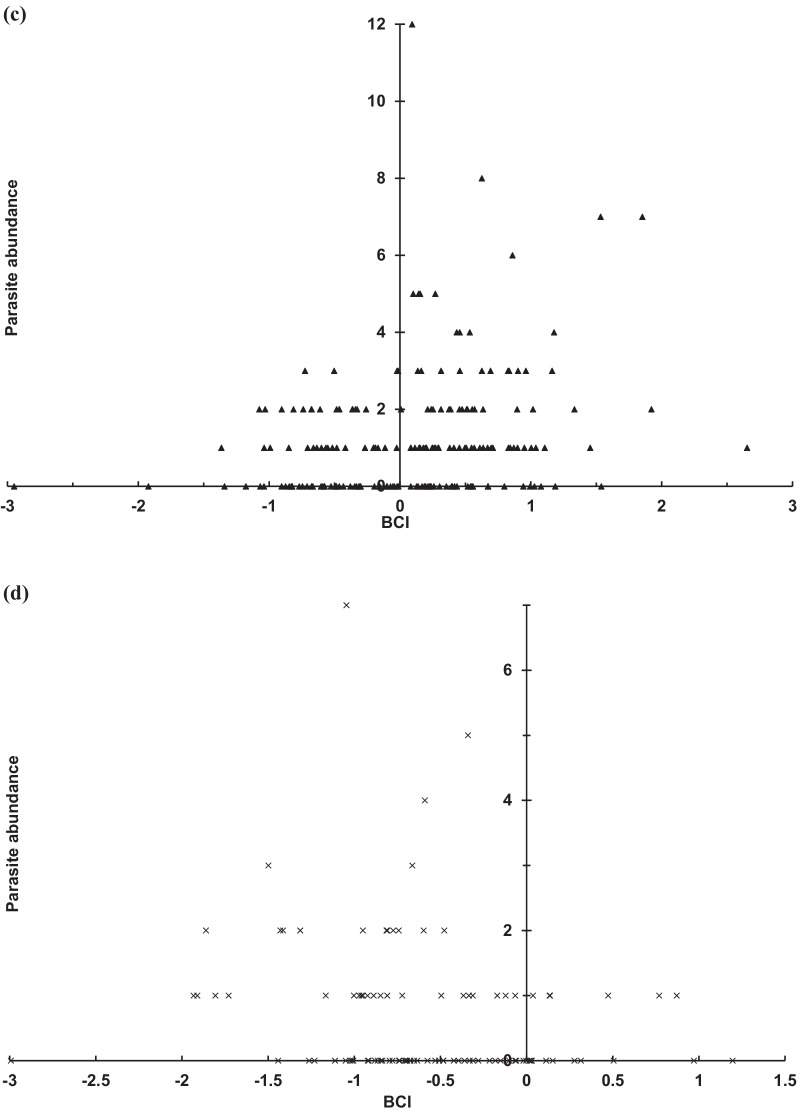
Abundance distribution of *Nycteribia allotopa* bat fly in relation to BCI values of **a** non-reproductive female (open circle), **b** reproductive female (filled circle), **c** male (filled triangel), and **d** juvenile (cross mark) bent-winged bats (χ^2^ = 42.7, *p* < 0.001) in the wet season in the GEF forest, Kenting, Taiwan

**Fig. 5:**
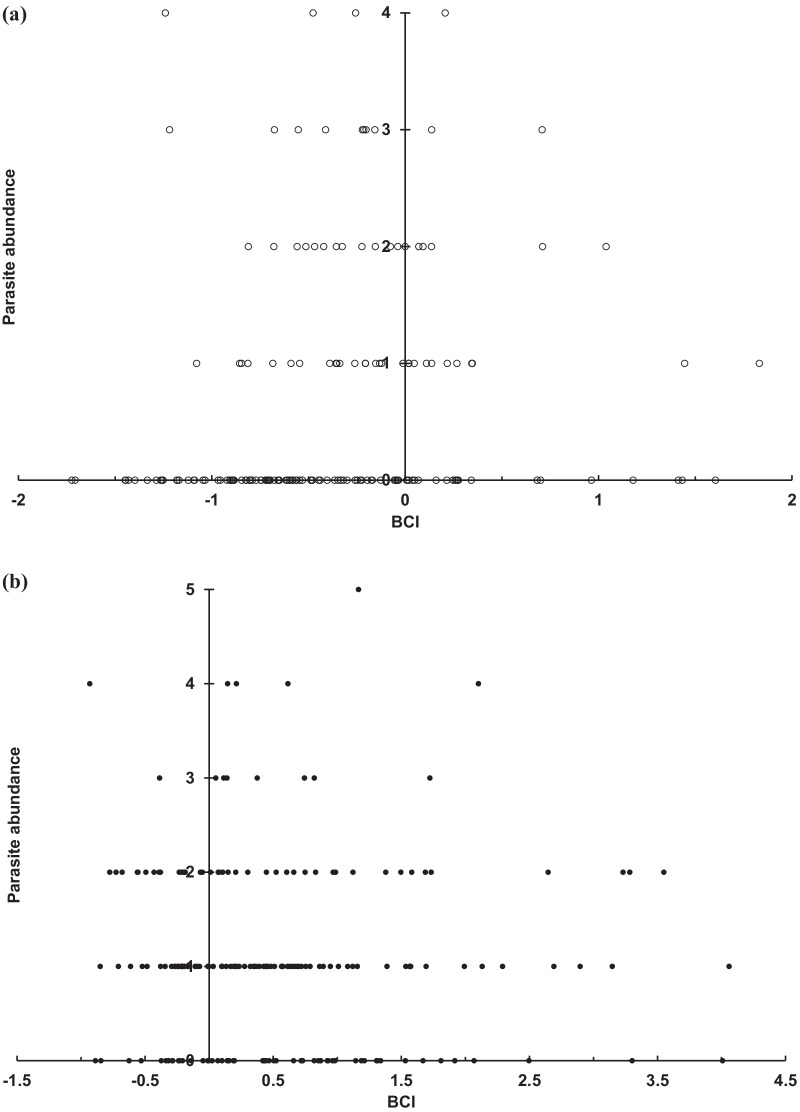

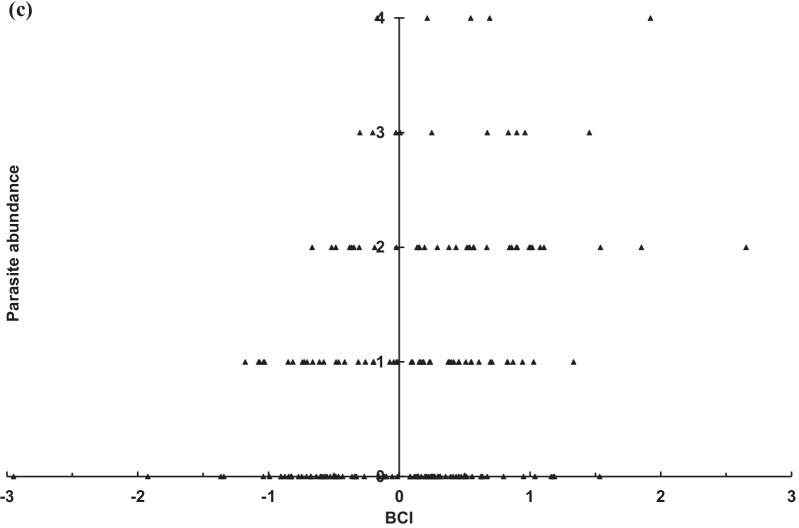
Abundance distribution of *Ascodipteron speiserianum* bat fly in relation to BCI values of **a** non-reproductive female (open circle), **b** reproductive female (filled circle), and **c** male (filled triangle) bent-winged bats (χ^2^ = 9.46, *p* < 0.005) in the wet season in the GEF forest, Kenting, Taiwan

Bat BCI values covaried with the infestation abundance of *N. allotopa* (Fig. 4) and *A*. *speiserianum* (Fig. 5). For both bat flies, the parasite abundance was generally distributed convexly around the medium to higher BCI values of the different bat groups, notably in reproductive females and males (Figs. 4b, c, and 5b, c). The bat fly *A*. *speiserianum,* however, was not found on juvenile bats and its parasite abundance ranged more concentratedly (Fig. 5), than that of *N. allotopa* (Fig. 4).

## Discussion

Our study reveals a high prevalence of multiple species infestation on the sampled bats, which has been rarely reported for nycteribiid bat flies in the Oriental tropical region [17, 21]. The bat fly abundances were generally higher on females, particularly reproductive females, than on males (Figs. 2, 3). The abundance of the bat flies *N. allotopa* and *A*. *speiserianum* on the bat *M. fuliginous* tended slightly towards bats of moderate or higher body condition (BCI), particularly reproductive females and adult males in the wet season (Figs. 4, 5). These results generally support our hypotheses regarding the effects of the body condition and host state on the parasite infestation. These results also concur with previous findings that bats with better body condition or nutritional status tend to be more heavily infested [10, 28, 35], and thus do not support the prediction of the tasty chick hypothesis [14]. However, in the present study, the relationship of the parasite abundance and body condition varies among different parasite species or across bats of various states in different seasons. This can be seen in the findings for the fly *A*. *speiserianum* infestation in the dry versus wet seasons (Fig. 1), and in the difference in the abundance of *A*. *speiserianum* and *N. allotopa* on non-reproductive female bats in the wet season (Figs. 4, 5).

Body condition of adult males was generally higher and less variable than that of non-reproductive females, but was lower than that of reproductive females (Table 1). Adult males, however, were less heavily infested than the adult females in the dry winter season, when females were not in reproductive status, or than reproductive females in the wet season (Figs. 2, 3). This finding is consistent with female-biased parasitism reported previously in bats [27–29, 31], and suggests that body condition alone may not be consistently sufficient to account for differences in the parasite abundance observed between sexes [55].

Bats, a true-flying mammal, rely predominately on sustained powered flight for moving and foraging. Thus, the eco-morphological and mechanical constraints imposed by the flight-related wing parameters (i.e., the wing loading and aspect ratio) presumably place upper limits on how heavy an individual can become, and limits on the variability of BCIs [56]. In addition, the difference in the immune defense capability of hosts with varying BCIs may not be consistently correlated or sufficient in itself to account for differences in the observed parasite abundance [57]. For instance, in bats lymphocyte and neutrophil proportions are generally similar irrespective of their body mass [58], which may explain why bats with lower BCI may not be more vulnerable to parasites than those in better condition [10].

Hormonal-immunological mechanisms contributing to sex-biased arthropod infestation in vertebrates are inconsistent and less studied (see review in [23]). Presumably the present findings of a higher parasite abundance on reproductive females in the wet season may partly result from their increased mass (due to fetal development), enhanced physiological stress, or greater parasite exposure in colonies during the breeding period [59]. Female mammals are often immuno-compromised when pregnant to avoid rejecting their pups [33]. Such suppression of the immune system, together with the natural physiological stress associated with pregnancy, may make reproductive females more vulnerable to infection, and thus contribute to female-biased parasitism [60–62].

In the present study, the lactating and post-lactating bats were heavier with higher BCIs than non-breeding females and were more heavily parasitized than both the non-breeding females and the males. Lactating females require energy for milk production [34, 63] and nonetheless, in the present study, lactating females showed the highest BCI of all the sampled bats. This suggests they must be foraging more actively to compensate for the increased energy demands of lactation and parental care [34, 64]. Post-lactating females had a lower body mass and BCI than lactating females, suggesting less foraging presumably resulting from a release from this energy constraint after juvenile weaning. This assertion is consistent with earlier reports that females increase their energy intake and accumulate energy reserves during pregnancy-lactation period, and then consume these reserves gradually as their offspring grow and develop [65]. Because of the time and energy required for foraging and nursing young, lactating females may reduce self-grooming, an effective but energetically costly anti-parasite behavior [66, 67], which could also help explain higher parasite abundance of lactating females observed here.

Juveniles after weaning may be more heavily infested with parasites than adults, because they are typically in poorer body condition due to their impaired flying and echolocation skills that lead to foraging performance [68]. In the present study, the juveniles showed the lowest bat fly abundance of all the bats (with the exception of the *P. jenynsii* flies, for which the abundance was higher than that of the males). However, they showed infestation abundance of mites second only to that of the reproductive females. Nycteribiid bat flies are generally agile and fast-moving [21], whereas mites are not [69]. Thus, the high mite abundance of the juveniles may stem from their close association with the mother during the lactation period [35]. An increased host density, and the close contact between mothers and their newborn pups vulnerable to parasites, may also account for the higher parasite abundances on lactating and post-lactating female bats [18].

The present findings supporting the proposed hypotheses that body condition and female reproductive status positively affecting parasite infestation are associated with reproduction related changes in the body condition and behavior of the hosts. Female bats, however, may be also preferentially infested by parasites even outside the breeding season [20, 27, 36]. The seasonality of reproductive bats typically coincides with the warmer months of the year [70], which are presumably favorable for parasites [71, 72]. The bats in the present study were sampled from southern Taiwan, a region characterized by tropical weather with limited variation in the temperature and humidity, and a relatively stable microclimate within the bat roost throughout the entire year. Our results therefore decouple the effects of host characteristics and weather factors, and indicate that warmer weather generally enhances parasite infestation in bats. In temperate areas, female bats enter hibernation with greater fat reserves, and consume the reserves more slowly than males and young of the year, which enhances the reproductive success of the females in the following spring [73]. The eastern bent-wing bats in southern Kyushu, Japan, hibernate as typical temperate bats [74]. The bats in our study area do not enter true hibernation in winter, but do become lethargic with reduced foraging activity in exceptionally cold and lengthy windy period [43, YF Lee unpub. data]. They also show similar reproductive patterns as the temperate conspecific [74]. This physiological effect could help explain why the females in the present study were also more infested than the males in the winter, if the change in fat reserves is also the case for female bent-wing bats in southern Taiwan, even in a lesser extent than that of temperate bats.

Although our results show a general trend toward female-biased infestation, the parasite species differ in their individual infestation distribution. Unlike wing mites that depend on contact for transmission, the agility, mobility, and reproductive characteristics of bat flies make switching among hosts much easier [21, 69]. It is likely that bat flies may actively select among hosts [20], which concurs with the lower prevalence observed in our study for these bat flies. Intriguingly, of the parasites with the lowest prevalence and abundance, the peculiar *A. speiserianum* flies are the heaviest and hardly move while infesting a host because the females embed themselves under the skin surface at the back of the host’s ear shell like cysts, whereas males are free ranging and rarely found [44]. Nonetheless, the difference in transmission modes of the bat flies and mites helps explain the observed discrepancies in previous studies: the mostly positive relationship between the host body condition (or mass) and the bat fly parasite infestation (e.g., [28, 35, 75]), but the mostly inconsistent pattern reported for mites [10, 36–39, 69, 76]. On the other hand, many species of bent-wing bats are infested by bat flies (e.g., *Nycteribia* spp., *Penicillidia* spp.) that themselves are parasitized by microparasites like fungi, bacteria, and blood parasites [77], but we still know little about ectoparasites as pathogen vectors for their bat hosts. Mobile bat flies vectoring pathogens may affect their hosts even later switching among bats, and can enhance pathogen transmission. Therefore, their negative effects on bat hosts may not be fully appreciated without more detailed sampling and monitoring of the same hosts, including recording their disease status, over a longer period, which would be worthy of further exploration.

## Conclusion

Our study reveals a generally female-biased, but parasite species-specific infestation on a widely distributed bat *M. fuliginosus* in the Oriental tropical region. Among the ectoparasites*, P. jenynsii* and *N. parvula* flies showed nearly consistent female-biased infestation throughout the entire year independent of host body condition. By contrast, the infestation of *N. allotopa* and *A. speiserianum* flies was affected by host body condition and also by host’s reproductive state in the wet season, but not in the winter, whereas the most prevalent wing mites showed only the effect of host state in the wet season. The results indicate a multi-parasite infestation associated with complex nested parasite distribution, where the life history, mobility and transmission mode, and potential interspecific interactions of ectoparasites [78, 79] and that of ectoparasites with their microparasites [77] may act integrally and interactively with the defense of the bat hosts.

## Data Availability

Not applicable.
